# Auscultation and Percussion

**Published:** 1855-03

**Authors:** 


Art. III.—*Auscultation and Percussion.* By Dr. Joseph Skoda. Translated from the fourth edition; by W. O. Markham, M. D., Assistant Physician to St. Mary's Hospital. Philadelphia. Lindsay & Blakiston. 1854. 8vo., pp. 380.

While the inestimable contributions of Laennec have done more towards the elucidatiou of chest disease than the combined labors of all his predecessors and successors, the authority of his great name has tended, in a most marked degree, to the perpetuation of some cardinal errors.

Few auscultators have ventured to differ with the founder of the science of Physical Diagnosis. The large majority have been content to accept, without so much as a question, his entire system in its fullest and most complete form. They have endeavored to reconcile the discrepancies they may have heard in the signs at the bedside and those described in the books, by rejecting the evidence of their own ears; and have labored to harmonize the most apparent contradictions and be satisfied with the most unsatisfactory theories and explanations rather than take a stand against the tenets of the illustrious Frenchman.

Ever since the appearance of LAENNEC's treatise, the tendency among men has been to force from the physical exploration of diseases of the chest consequences which do not legitimately flow from it. But the number of observers who now-a-days boast, and teach others to believe, that auscultation can demonstrate the hidden workings of the organs of the chest, with something of the same degree of certainty as if they were the direct objects of vision, is happily growing less, and will, it is to be hoped, soon wholly disappear. The publication of such works as that now under review can but tend to hasten this result.

As we before had occasion to remark, in a previons number of this journal, no one can have adequately studied the subject of physical examination of diseases of the chest, without feeling that a vast amount of difficulty and obscurity is still attached to it, and "that we have much to learn, and perhaps much to unlearn, before we can hope for anything like perfection, in our diagnosis of these diseases." With all its vaunted certainty, few, we imagine, can be found sufficiently bold to deny that "the most skillful and practised observer has often to do penance for his errors, before facts revealed by the knife of the anatomist."

SKODA, who is an acknowledged master of his subject, and, from all accounts, unrivalled in his powers of diagnosis, never lets an opportunity pass of warning his readers when they have in any particular case obtained all the information which auscultation can afford them, still, before they conclude their diagnosis, to pause, and to lay hand upon every other aid which other sources can supply. This warning of the Viennese professor has too often been made the text of a flout at physical diagnosis by those whose industry or opportunities have not been sufficient to make them acquainted with even the simplest truths of the science.

An incubus which hovers over the very threshold of the study of auscultation, and which, perhaps, has tended more than any other one thing to impede its progress, is the vagueness and diversity, and as Dr. MARKHAM aptly remarks, the repulsiveness of the terms employed by different authors to indicate its phenomena. Dr. LATHAM directed attention to this subject a good many years ago. "Auscultation," he says, "is capable, I have thought, of being greatly simplified for practical purposes. At all events, unless it be so, it can never be successfully taught; the knowledge derived from it must be confined to a few physicians of hospitals, and the profession at large can never expect much benefit from it." He even went so far as to substitute, to some extent, Saxon for Gallic terms. But there appears to be an attractiveness in the over-refined nomenclature and minutiæ of the French school which has quite eclipsed the every day English garb in which he proposed to clothe auscultatory phenomena. The result has been a deplorable amount of confusion in this department of medical literature, so great indeed, that the idea conveyed to the mind of the reader is frequently different from that intended by the writer. And it will thus continue so long as authors use the same terms in different senses. "How," Dr. MARKHAM remarks, "is it possible that precise ideas of the value of a particular sign can fix themselves in the student's mind, when the sign is represented to him by different authors under such a variety of terms? Here, for instance, are a few of those by which the râle crépitant humide of LAENNEC is known amongst us: moist crepitantronchus, crepitation, crepitating râle, crepitant ronchus, crepitant râle, minute crepitations, vesicular râle, crackling of pneumonia, small crepitations, râle sous crépitant du catarrhe pulmonaire aigu capillaire." Dr. THEOPHILUS THOMPSON, in his lectures on Pulmonary Consumption, has proposed a modification of the nomenclature of auscultation which does not, to our minds at least, add anything to its simplicity; and being a classification of the sounds according to the stages and processes of diseases which produce them is manifestly, according to the best authorities, erroneous. SKODA has also made innovations in this direction, but unfortunately, we think, he has based them upon his theory of consonance. And although none can deny that his nomenclature is eminently plain and to the purpose, its adoption, as his translator remarks, can hardly be hoped for until such time, at least, as his theory has met with more general approbation.

The opening chapter of SKODA's work is on percussion. Neither AUENBRUGGER, CORVISART, nor LAENNEC used a pleximeter in percussion. The introduction of this instrument is due to PIORRY. SKODA uses the ivory dirk, known as the pleximeter of PIORRY, and strikes with the fingers. LOUIS prefers a pleximeter made of caoutchouc. SKODA thinks it does not produce so clear a sound as the instrument he uses. The pleximeter, however, whether constructed of ivory, caoutchouc, or leather and whether struck with the finger or with a steel hammer possesses in point of clearness of the sound elicited very slight if any advantages over the finger; but whoever practices percussion extensively will prefer the pleximeter, on account of the pain occasioned by constant percussion of the finger. PIORRY and his pupil MAILLIOT LEON, with their national fondness for almost endless and infinite division and subdivision of subjects, affirm that there is a liver, spleen, kidney, heart, lung and stomach sound. SKODA says there is no difference in the percussion sound by which we can distinguish between organs not containing air; a hard liver yielding the same sound as a soft liver, a hard spleen as a soft spleen, and blood the same sound as pus, water, &c. LAENNEC divided percussion sounds into clear and dull. SKODA distinguishes four varieties, the extremes of which are represented by the following terms:—

1. Full—Empty.
2. Clear—Dull.
3. Tympanitic—Non-tympanitic.
4. High—Low.

A full percussion sound may be clear or dull, high or low, tympanitic or non-tympanitic. The full sound characterises the size of bodies, is persistent, and spread, as it were, over a large surface; the less full, or empty sound is the reverse of this. The stomach distended with air yields a full, the small intestines an empty sound. SKODA believes that the latter sound heard at any yielding part of the walls of the thorax, or the abdomen, indicates that no air is present in that part, for a space of several inches in depth, and one inch or more in circumference.

We have not met with the terms *full* and *empty* in any other author, and we cannot, for our part, see any advantage which is to follow their introduction into the nomenclature of percussion sounds. It but adds another difficulty to a subject which all agree is already sufficiently complex. We even venture the opinion that they cannot be of much practical utility and that the number of practitioners who will be able to distinguish, for instance, between a clear full and a clear empty, or a dull empty and a dull full sound, will be very small.

The terms *clear* and *dull* are to be taken in their usual signification, and these are regarded by most auscultators in this country as a sufficient division of percussion sounds for all practical purposes.

The tympanitic sound is not thought by SKODA to depend on the quantity of air an organ contains, but rather on the amount of tension of its parietes. The percussion sound is invariably tympanitic when the parietes of the organ which contains the air are not stretched; when, on the other hand, they are firmly stretched, the percussion sound becomes less or not at all tympanitic, and even dull.

The relaxed stomach, the compressible abdominal walls, the collapsed lungs, give a distinctly tympanitic sound. The fully distended stomach, the firmly contracted abdominal walls, the tense thorax, the strongly inflated lungs produce a non-tympanitic or merely an indistinctly timpanitic sound. "A greater homogeneity of vibrations appears necessary for the production of a tympanitic, than of a non-tympanitic sound. When percussion is made upon a non-distended stomach, it is the air alone within it which produces the sound; but if the stomach be strongly distended, its coats also vibrate, and these vibrations seem to interfere with those of the contained air, and thus to be the cause of the dull non-tympanic sound."

SKODA regards the *high* and *low* sounds as of little value in practice.

The Metallic Ringing Percussion sound, and the Cracked-pot sound cannot be classed in any of the foregoing divisions. PIORRY believes that the presence of water and air together is necessary to produce the first sound. SKODA denies that the presence of water is required, and in proof of his position refers to the demonstration afforded by percussing a stomach filled with air and not containing one drop of water; this experiment succeeds best when the coats of the stomach are not made too tense. The sound may however, be heard when the stomach contains both air and water.

SKODA says he has never observed the cracked-pot sound in children whose lungs contained no cavities.

LAENNEC, as is known, attributed the variations observed in the strength and clearness of the thoracic voice to changes in the structure of the lungs either by infiltration and consolidation or by the presence of fluids in the pleura. SKODA takes ground against this theory and declares that repeated experiments have convinced him that sound is heard somewhat further through healthy than through hepatized lung. He deems the differences in the sound conducting power of healthy and diseased lung substance as insufficient to explain the variations in the thoracic voice and opposes LAENNEC's views on the mechanism of bronchophony in particular, on the following grounds: that, first, we find in hepatized lung that bronchophony may in the course of a few minutes appear and disappear without the other physical signs having undergone any change; secondly, when vocal resonance is temporarily absent, it may be restored by the patient's breathing deeply or coughing, thereby clearing the bronchi in the hepatized part; thirdly, if a healthy and a solidified lung be taken from the body and while one person directs his voice into a stethescope placed upon each organ successively, a second listens through another stethescope, it will be found that the resonance is heard somewhat further through healthy than through hepatized lung; fourthly, in the course of pleuritic effusions, the voice becomes weaker in proportion as the effusion progresses—the reverse of which should occur if LAENNEC's theory were correct. SKODA affirms that the human voice and every other sound which is formed or propagated in the air, is heard furthest in the air. "A sound excited in the air, is heard very indistinctly, or not at all, by a person under water; and a sound in one room passes with difficulty into another, being interrupted by the walls. Any one wishing to weaken his hearing, stops his ears.

"On the other hand, the slightest scratching at one end of a long rod may be heard, if the ear be brought in contact with the other; while no sound whatever is audible in the ear, although the ear be brought much nearer to that end of the rod whence the sound proceeds. The sound caused by striking two stones together under water, is distinctly heard there, and even causes a disagreeable sensation; whilst, out of the water it can be scarcely recognized.

"These facts show that sound does not pass readily from dense bodies into the air, or from the air into dense bodies. Physics, also, teach us that sound is always reflected, in passing from one medium into another, and that less sound enters into the new medium than would have been propagated through a corresponding space of the one in which it was originally excited; the more dissimilar the media are, in respect of density and cohesion, the greater is the reflection of the sound, and the less freely does it pass from the one into the other."

Skoda believes that bronchophony is produced by consonance of the air, within the bronchial tubes, with the laryngeal voice and denies that the walls of the bronchi and trachea play any part in the conduction of the sonorous vibrations of the vocal chords. It is with this theory that the name of Skoda is most generally associated in this country. We cannot do better perhaps, than to copy the sketch of it in the language of Dr. Markham:—

"The voice passes into the parenchyma of the lungs through the medium of the air in the trachea and the bronchial tubes, and is not propagated along the walls; it traverses healthy, as readily as it does hepatized lung, and even somewhat more readily: consequently, bronchophony does not depend upon an increase of the sound-conducting power of consolidated pulmonary tissue; moreover, when the lung is consolidated, the thoracic voice increases and diminishes in force, without any concurrent change taking place in the condition of the lung; this variation in its strength evidently results from the circumstance of the bronchial tubes being at one moment blocked up by mucus, etc., and at another freed therefrom by the cough and expectoration, etc.; if the bronchophony depended upon conduction of sound, it would be a matter of indifference whether the tubes contained air or fluids. It must not be forgotten, that, according to the ordinary laws of reflection of sound, the more solid the parenchyma the more difficult does the passage of the sound from the air into it become.

"That the air in the mouth and nasal cavities consonates with sounds formed in the larynx, is proved by the fact of the changes which the voice undergoes through opening and closing the mouth and nose, whilst the condition of the larynx remains unaltered; just in the same way does the air in the trachea and bronchial tubes consonate with the laryngeal sounds. Now, air consonates only in a confined space, and the force of the consonance depends upon the form and size of the space, and upon the nature of the walls forming it; the more solid the walls, the more completely will the sound be reflected, and the more forbible the consonance. The cause of the loud voice produced by a speaking-trumpet is well-known. But the air will consonate with certain sounds only; in the trachea and bronchial tubes, it becomes consonant with the laryngeal voice, in so far as their walls have a like or an analogous character to the walls of the larynx, of the mouth, and of the nose. Within the cartilaginous walls of the trachea and the bronchial trunks, the voice consonates nearly as forcibly as in the larynx; but as the bronchial tubes divide in the lungs, they lose their cartilaginous character, becoming at last merely membranous in structure, and therefore very ill-adapted for consonance; when, therefore, the consonance is increased in these latter tubes, we may be sure, either that the membrane forming them has become very dense or cartilaginous, or that the tissue arouud them is condensed and deprived of air, whereby the sound-reflecting power of the tubes is increased. Of course the communication between the air in the tubes and the air in the larynx must be uninterrupted.

The walls of a confined space frequently vibrate in unison with sounds excited within it, as do those of an organ pipe, or of a speaking-trumpet. The larynx vibrates with every sound, and its vibrations are perceptible at a considerable distance from their point of origin; so, also, must the walls of the bronchial tubes, which are distributed through the parenchyma of the lungs, vibrate when the voice consonates within them; and the vibrations thus excited will extend to the surface of the thorax, passing through several inches of thick fleshy parts, or of fluids, and manifest themselves there as the consonating sounds of the bronchial tubes."

While the perfect passage of the voice through the stethescope occurs, according to Laennec, only in perfect pectoriloquy, yet even he was obliged to admit that pectoriloquy could not be distinguished from bronchophony without taking into consideration the situation and extent of the resonance, the nature of the cough, the respiration, the râles, &c. He has laid down no distinctive sign between the two conditions of voice just named; calling at one time the same voice pectoriloquy, and at another bronchophony, according as it is heard in different situations and over different extents of surface, and is accompanied by certain phenomena derivable from the respiration, the râles, and the disturbance of the general functions.

How are we, SKODA asks, to ascertain from the nature of the voice, whether the resonance heard proceeds from a cavity, or from a bronchial tube? He justly considers the question not answered by simply calling the resonance of the voice in cavities pectoriloquy, and that in bronchial tubes bronchophony, without pointing out any essential difference between the two, and deems the attempt to draw a distinction between pectoriloquy and bronchophony altogether useless and only productive of error.

LAENNEC regarded ægophony as the natural resonance of the voice in the bronchial ramifications which are flattened and compressed by fluid in the pleura; the resonance traversing a thin tremulous layer of fluid and becoming audible as the result of the substance of the lung being compressed and denser than natural and thus made a better conductor of sound.

SKODA says he cannot, in accordance with his experience, admit ægophony to be a sign of pleuritic effusion. He declares that he has met with this voice both when fluid existed in the pleura, and when no trace of it could be found there; also in pneumonia and in tubercular infiltrations, with or without cavities; and he has also observed that in pleuritic effusions as well as in pneumonia unattended by such effusion, single words or syllables presented the tremulous, bleating character of the voice, whilst other words were entirely free from it; indeed, this sound has been heard, both by himself and others, over the inter-scapular region in women and children, whose lungs were entirely healthy.

SKODA does not believe that flattening of the bronchial tubes has anything to do with the production of the tremulous resonance. He thinks that a tremulous sound can only be produced by the impact of one solid body upon another, or upon some fluid or aeriform body, and that mere vibrations of the air cannot give rise to it. "Such impact, however, can only occur when the voice consonates in some air-containing space within the thorax; for as we have already shown, vibrations are not communicated from the larynx to the parenchyma of the lungs along the walls of the trachea and the bronchial tubes. It is therefore probable that in most cases the walls of the bronchial tube, within which the air consonates, react by impact on the air contained within them, and so give rise to the tremulous sound. It is possible however, that it may be occasionally produced by a portion of mucus, etc., partially closing the mouth of the bronchial tube, imitating the thin tongue in the mouth-piece of tongued instruments."

SKODA, as we have endeavored to show, assumes that LAENNEC's pectoriloquy and bronchophony represent one and the same phenomenon; and that ægophony has no necessary connexion with the presence of fluid in the pleura, nor, as a sign, any particular value.

He, on the other hand, recognises four modifications of the thoracic voice:—Loud bronchophony, weak bronchophony—an indistinct humming, amphoric resonance and the metallic echo of the voice.

If we understand the two authors, LAENNEC and SKODA, we can see no difference between the pectoriloquy of the former and the loud bronchophony of the latter. The definition of the two sounds as laid down by the Parisian and Viennese professors are as follows:—

LAENNEC. { *Pectoriloquy.* The voice passes wholly through the stethescope.

SKODA. { *Loud Bronchophony.* The voice accompanied by a concussion on the ear, completely traverses the stethescope.

SKODA attaches no great importance to resonance of the voice alone, except when it presents itself as undoubted bronchophony. If bronchophony be not sufficiently loud or clear to warrant positive conclusions, our author says, we may yet obtain some tolerably safe results, by comparing it at different parts of the thorax, especially at corresponding parts of the two sides. But we are again charged to take into consideration every sign which auscultation and percussion can afford us, before we come to any decided conclusion.

LAENNEC and his disciples, among whom as especially prominent may be mentioned, BARTH and ROGER, FOURNET and WALSHE, considered the clear whispering thoracic voice as a variety of bronchophony and esteemed it well nigh pathognomonic of the existence of a cavity. SKODA says that when the clear whispering is unaccompanied by amphoric resonance or metallic tinkling it is of no more value as a cavern sign than any other vocal sound; at the same time he declares it is positive evidence of the existence of some disease of the respiratory organs.

The doctrine of autophonia, first announced, we believe, by Dr. HOUSMANN, and which at one time created some discussion among observers, our author thinks entirely valueless as a sign of disease. He says it will be found that if the auscultator himself speak while examining a patient, bronchophony will invariably be heard whether the lung be healthy or diseased.

SKODA attaches no distinct signification to the indistinct humming, nor to a complete absence of all resonance of the voice. Humming may be observed not only in the normal state of the lungs, but may be met with in any of their diseased conditions. And if this be so, we can see no reason why so indefinite a phenomenon should be retained in the list of voice changes.

Under the head of murmurs caused by the motion of the air during respiration, our author distinguishes respiratory murmurs, râles, whistling and sonorous sounds. The three latter are caused by the presence of fluid in the air-passages, by thickening of the bronchial mucous membrane, and by any partial narrowing or compression of the bronchial tubes.

The respiratory murmur in children is much more distinct and loud than in adults, while there is no difference in the intensity of their laryngeal murmur. Hence it may be concluded that the murmur as heard in children, presents the most perfect characters proper to the respiratory murmur of the air cells and finer bronchial tubes. The character of the different *respiratory murmurs* may be imitated by drawing air into, or forcing it out of the mouth, whilst the lips are placed in the position which is requisite for the conversion of an unarticulated into an articulated laryngeal sound, or in other words the pronunciation of a consonant and a vowel. That position of the mouth which is necessary for the pronunciation of any particular consonant and vowel, always gives rise to the same murmur.

The laryngeal, tracheal, and bronchial murmurs may be imitated by forcing air against the hard palate; this is done involuntarily in hard breathing. The consonant which answers to these murmurs is *ch* guttural, or it falls between *h* and *ch*.

The murmur of the cells and finer bronchial tubes may be imitated by narrowing the opening of the mouth, and then drawing in the air. The consonant of this murmur is *v* or *b*. The expiratory murmur differs from the murmur of inspiration and resembles rather a gentle aspiration, or blowing. The consonant which corresponds to it falls between *f* and *h*.

It is evident from what has been said that SKODA believes the larnygeal, tracheal, and bronchial respiratory murmurs may, like the thoracic voice, be increased by consonance within the thorax. It is hardly necessary to add that the conditions necessary to produce consonance of these murmurs, are the same as those required for the voice.

SKODA differs from LAENNEC in regard to the value of the weak and indistinct respiratory sound. The German professor believes that the respiratory murmur may be heard of equal strength and clearness over the whole of the chest, "even when considerable portions of the lung are distended and congested, and that it may be strong at one part, and weak and indistinct at another, when there exists *no abnormal affection of the lungs, which is appreciable.*" The italics are our own.

The explanation which LAENNEC gives of the cause of the bronchial respiration is that which has been most generally accepted by observers. SKODA does not consider it correct. His belief is that the bronchial respiration, like the bronchial voice, must be explained by the laws of consonance. The conditions which develop the one will also give rise to the other. He further regards LAENNEC's bronchial and cavernous respiration as one and the same murmur, and holds that there are respiratory murmurs heard over the chest, which cannot be classed under the head either of bronchial or of vesicular murmurs.

Our author divides respiratory murmurs as follows:—Vesicular breathing,(ANDRAL),—Bronchial breathing,—Amphoric echo and metallic tinkling heard during breathing,—Indeterminate respiratory murmurs. D. W. Y.

(*To be Continued.*)

